# Drivers and consequences of child marriage in a context of protracted displacement: a qualitative study among Syrian refugees in Egypt

**DOI:** 10.1186/s12889-021-10718-8

**Published:** 2021-04-07

**Authors:** Shatha Elnakib, Salma Abou Hussein, Sali Hafez, May Elsallab, Kara Hunersen, Janna Metzler, W. Courtland Robinson

**Affiliations:** 1 Johns Hopkins Bloomberg School of Public Health, Baltimore, USA; 2 Independent consultant, Cairo, Egypt; 3 UNFPA, Cairo, Egypt; 4 Women’s Refugee Commission, New York, USA

**Keywords:** Displacement, Humanitarian context, Child marriage, Adolescent health, Sexual and reproductive health, Syrian refugees, Egypt

## Abstract

**Background:**

Child marriage is a human rights violation disproportionately impacting girls in low- and middle-income countries. In the Middle East region, conflict and displacement have prompted concerns that families are increasingly resorting to child marriage to cope with economic insecurity and fears from sexual violence. This study set out to examine child marriage among Syrian refugees residing in Egypt with the aim of understanding drivers of child marriage in this context of displacement as well as how child marriage affects refugee girls’ wellbeing.

**Methods:**

This analysis draws from 15 focus group discussions (FGD) conducted with married and unmarried girls, as well as parents of adolescent girls in three governorates in Egypt. FGDs included a participatory ranking exercise and photo-elicitation. Additionally, we conducted 29 in-depth interviews with girls and mothers, as well as 28 key informant interviews with health providers, community leaders, and humanitarian actors. The data was thematically analyzed using a combination of inductive and deductive coding.

**Results:**

A prevalent phenomenon in pre-war Syria, child marriage has been sustained after the influx of Syrian refugees into Egypt by pre-existing cultural traditions and gender norms that prioritize the role of girls as wives and mothers. However, displacement into Egypt engendered different responses. For some families, displacement-specific challenges such as disruptions to girls’ education, protection concerns, and livelihood insecurity were found to exacerbate girls’ vulnerability to child marriage. For others, however, displacement into urban areas in Egypt may have contributed to the erosion of social norms that favored child marriage, leading to marriage postponement. Among girls who were married early, we identified a range of negative health and social consequences, including lack of family planning use, disruption to schooling and curtailment of girls’ mobility as well as challenges with marriage and birth registration which accentuated their vulnerability.

**Conclusion:**

Efforts to address child marriage among Syrian refugees must acknowledge the different ways in which displacement can influence child marriage attitudes and practices and should capitalize on positive changes that have the potential to catalyze social norm change. Moreover, targeted, focused and contextualized interventions should not only focus on preventing child marriage but also on mitigating its impacts.

**Supplementary Information:**

The online version contains supplementary material available at 10.1186/s12889-021-10718-8.

## Background

Ending child marriage has received increased attention over the past decade and is now a global priority, as highlighted in Sustainable Development Goal 5 which aims to achieve gender equality and eliminate harmful practices. Yet, while the practice is declining worldwide, it remains widespread in many parts of the world with an estimated 40% of girls in the least developed countries marrying before age 18 [1].

There is a large body of literature linking child marriage to a wide range of adverse consequences for young married women and their children. Much of this literature focuses on girls’ increased risk of negative sexual and reproductive health outcomes, such as early and unintended childbearing [2, 3], low contraceptive use, and high fertility [2, 4, 5]. Several studies indicate that girls married as children have a higher propensity for negative mental health outcomes, such as depression and anxiety [6], lower self-efficacy [7], and stress [7, 8]. Moreover, child marriage has been linked with marital rape and lack of involvement in spouse selection [9–12], lower levels of autonomy and decision-making [13, 14], and overall poor marriage quality [15, 16]. Several studies have also linked child marriage with negative neonatal and child health outcomes, including an increased risk of mortality for children born to young mothers [7, 11, 17–19].

In the Middle East region, considerable declines in child marriage rates took place from 1985 to 2010, but progress has more or less stagnated over the last 10 years [20, 21]. Nonetheless, prevalence of child marriage remains lower than other regions including South Asia, Sub-Saharan Africa and Latin America. As of 2015, 19% of women ages 20–24 in the Middle East were married before age 18 and 4% before 15 [21, 22]. However, the region’s prevalence estimates mask significant inter-country variation, with child marriage reaching a high of 30% in Yemen and a low of 2% in Tunisia [21].

The existence of multiple intractable and protracted conflicts in the region has prompted concerns that child marriage rates may have increased in recent years. Indeed, studies with Palestinians [23], Yemenis [24], and Iraqis [25] have documented increases in child marriage during conflict and in its aftermath. More recent empirical evidence from the Syria crisis further indicates that refugees in Lebanon and Jordan are increasingly practicing child marriage. A study conducted among Syrian refugees in Jordan demonstrated that while 12.0% of registered marriages in pre-war Syria involved a girl under the age of 18, 18.4% involved children in 2012, 25.0% in 2013 and 31.7% in the first quarter of 2014 [26]. Similarly, a national household survey undertaken in Lebanon in 2016 found that child marriage rates reached 40% in Syrian refugee communities [27].

Qualitative studies conducted on child marriage among Syrian refugees have helped provide an in-depth and contextualized understanding of social and cultural contexts and mechanisms underlying this practice. Studies conducted in Lebanon indicate that the combination of poverty, loss of educational opportunities, and heightened protection concerns – all resulting from displacement – pushed families to resort to child marriage as a coping mechanism [28–30]. These findings are echoed by another study which similarly finds that poverty, an overemphasis on *sutra* – a concept that denotes social and financial protection – as well as pronatalist motivations were major driving forces behind child marriage among Syrian refugees in Jordan [26].

Prior to the war, child marriage was not uncommon in Syria. In fact, until recently, personal status laws set out the minimum age of marriage at 17 for girls and 18 for boys, and allowed exceptions with approval from a judge [31]. Population-level estimates of child marriage prevalence in pre-war Syria are outdated, with the last Multiple Indicator Cluster Survey in 2006 suggesting that 13% of women ages 20–24 were married before age 18.

Now in its eighth year, the Syrian conflict has led to the displacement of over 5 million refugees to Lebanon, Turkey, Jordan, Iraq and Egypt. As of September 2019, around 130,000 refugees are officially registered in Egypt, half of whom are female and close to 20% are girls under age 18 [32]. While requirements introduced by the government in 2013 restrict entry of Syrians into Egypt, registered Syrian refugees are allowed to regularize and renew their residency. Egypt has not adopted a policy of encampment, and instead, refugees live in predominantly urban and peri-urban areas alongside Egyptian communities. The highest concentration of Syrian refugees is in Greater Cairo – which includes Cairo, Giza and Qalyubia – as well as Damietta and Alexandria. A presidential decree issued in 2012 granted Syrian refugees access to public schools and health care on par with Egyptian nationals.

While child marriage among Syrian refugees has been explored in the context of Jordan [26, 33], Turkey [34] and Lebanon [28, 29], child marriage has not been investigated in the context of the Syrian influx in Egypt [35]. To our knowledge, this is the first study examining child marriage practices among Syrian refugees in Egypt with the aim of understanding drivers of child marriage in this context of displacement as well as how child marriage affects refugee girls’ wellbeing. The study was part of a four-country study of child marriage in the Arab States Region carried out by the Women’s Refugee Commission and the Johns Hopkins Bloomberg School of Public Health, with support from and in local partnership with UNFPA and UNICEF. Other study sites were Djibouti, Yemen and Northern Iraq which included household surveys of adolescent girls and adult women as well as qualitative methods in Yemen and Northern Iraq (the Djibouti study involved only quantitative research). Due to restrictions on quantitative data collection in Egypt, we exclusively relied on qualitative data to deepen our understanding of drivers and consequences of this practice.

## Methods

### Study sites and participants

**Fig. 1:**
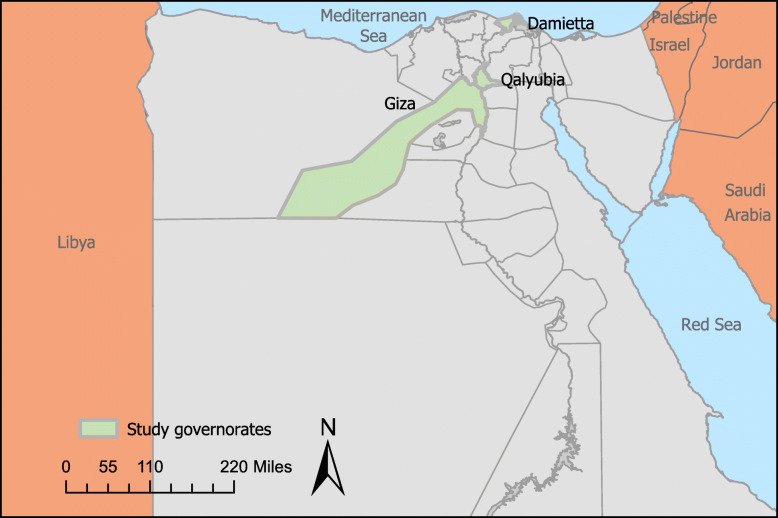
Map of Egypt with study sites highlighted in green, generated using ArcGIS Pro

The study took place in three purposively selected governorates in Egypt: Giza, Damietta, and Qalyubia (Fig. 1). The governorates were selected based on UNHCR estimates which indicate that the three governorates have high concentrations of Syrian refugees. They are also governorates in which UNFPA and UNICEF are actively involved.

Giza hosts a total of 36,728 Syrian refugees, Qalyubia hosts 17,905 and Damietta hosts 8807 [36]. Giza, which is part of Greater Cairo, is considered partially urban and is home to the biggest community of Syrian refugees, particularly in 6th of October city. The governorate of Qalyubia is also part of Greater Cairo and is largely rural. However, Syrian refugees predominantly reside in the industrial zone of El-Obour which houses a number of textiles, pharmaceutical, plastic and cement factories. Unlike the other two governorates, Damietta is a port city and is situated on the Mediterranean Sea. Although largely rural, Damietta has hosted Syrian refugees in its urban centers like Damietta City.

The analysis presented in this paper draws from (1) 15 focus group discussions (FGDs) – with participative ranking methodology and photo-elicitation, (2) 29 in-depth interviews (IDIs) with married and unmarried girls as well as mothers of adolescent girls, and (3) 28 semi-structured key informant interviews (KIIs) with humanitarian actors, health providers, legal experts and community leaders (Table 1). To be selected to participate in the study, married and unmarried girls had to be aged 10–19 and married girls had to be married under age 18. FGDs consisted of 5–10 participants each and were conducted with Syrian married and unmarried adolescent girls as well as mothers and fathers of adolescent girls. Among married girls, the mean age was 17.7 and mean age at marriage was 15.97; while the mean age of unmarried girls was 16.6. Most married girls had primary (48.4%) or middle school (45.16%) as their highest level of education and only 6.4% reported receiving a high school education; whereas 40% of unmarried girls had a high school education and 6.67% had gone on to university. Fathers were on average aged 48.4 years old and mothers were 41.6. Their mean ages at marriage were 27.4 and 18.01 respectively. Most fathers reported primary (33.3%) or middle school (44.44%) as their highest level of education and 22.2% had a high school or university education. Among mothers, 56.4% had primary or middle school education and the remaining 43.6% had a high school or university education.

**Table 1:** Number of IDIs, FGDs and KIIs by governorate

		Giza	Damietta	Qalyubia	Total
IDIs	Married Girls	3	5	3	11
	Unmarried Girls	3	3	3	9
	Mothers	3	3	3	9
FGDs	Married Girls	1	1	1	3
	Unmarried Girls	2	1	1	4
	Mothers	2	1	2	5
	Fathers	1	1	1	3
KIIs	Community Leaders	3	2	4	9
	Health Providers	2	2	2	6
	Humanitarians	Humanitarian actors were based in Cairo			11
	Legal experts	One Syrian lawyer and one Egyptian lawyer based in France and Egypt, respectively			2
Total		20	19	20	72

We used the databases of UNFPA Safe Spaces and UNICEF Family Centers as a starting point to recruit Syrian participants who were new users of UNFPA-and UNICEF- supported services, and who fulfilled preset eligibility criteria. Once participants were identified, they were contacted by phone or in-person by the UNFPA Safe Spaces staff / UNICEF Family Centers staff and read a recruitment script to confirm eligibility. To be eligible for an FGD/interview, participants had to be Syrian refugees or Egyptians who worked with Syrian refugees and who lived in the governorate at least one month in the last year. They also had to fulfill the age and location requirements noted above. Snowball sampling was then applied to identify additional individuals who fit the eligibility criteria and who had not received interventions or had very minimal contact with services. Key informants were selected based on suggestions from UNICEF, UNFPA and their local partners – Terre Des Hommes, Ministry of Youth and Sports, and Etijah. Second-wave participants were then selected via snowballing.

### Data collection

Data were collected between February 2019 to August 2019. Five Syrian and five Egyptian data collectors were recruited and trained in a six-day in-depth training – as well as a refresher training later in the study – on the research protocol, human subjects research, qualitative interviewing, critical and epistemological reflexivity, and conduct of participatory research. FGDs and IDIs were undertaken in UNFPA safe spaces, UNICEF family centers and primary health units, with the exception of one FGD which took place in the residence of a community leader. KIIs took place at safe spaces, health facilities, or the offices of participants. The first and second author conducted additional interviews with key informants. All data collection took place face-to-face with the exception of two key informant interviews with legal experts which took place via Skype.

FGDs were conducted by a team of one facilitator and one note-taker of the same gender and nationality as the participants, while IDIs and KIIs were undertaken by one data collector (also of the same gender and nationality). Notetakers were responsible for ensuring thorough documentation of verbal and non-verbal communication and collected participants’ sociodemographic data while facilitators obtained consent and moderated the discussion. All data collection with Syrian participants took place in Syrian-Arabic dialect. Data collectors used discussion guides which were developed specifically for the study in collaboration with UNFPA and UNICEF staff around key topics of programmatic relevance and of interest to the research team (All guides are available in supplementary files 1, 2, and 3). The discussion guides were additionally reviewed and refined after consultation with the local data collection team to ensure that questions were relevant and culturally appropriate. All tools were pre-tested before being used in the study.

### Photo-elicitation and participative ranking methodology

We drew on visual and participatory methods to generate insights and perceptions that are grounded in participants’ viewpoints and sociocultural context. The first technique that we used was participative ranking methodology (PRM) which falls under the broader research method of participatory rapid appraisal (PRA) [37]. This method uses the key steps involved in PRA: Pile, Rank, Account. By posing open-ended questions, facilitators elicit insights from participants and use sticky notes to jot down key themes emerging from the discussions. These are then ranked by participants according to their relative importance. Participants discuss the ranking until a final ordering is reached – at which point, the facilitator records the reasons provided by participants for their decisions and the ordering of the items. In the study, we used PRM in FGDs with mothers and fathers, asking them about what they believed were reasons why parents in their present community married their girls early.

We also employed photo-elicitation: a visual method that helps researchers understand the lived experience of participants. FGDs with married and unmarried girls incorporated this technique to elicit in-depth discussion and encourage participants to speak freely. Several studies have found that the technique allows for a better understanding of social phenomena and is particularly useful in public health research and research with children [38–40]. We used locally relevant pictures of married and unmarried girls – which we obtained from UNFPA and selected in consultation with the local team – and asked participants to describe what they see. Specifically, participants were asked if they think the girl is unmarried/married, and if they can describe her daily activities. If participants identified the picture as that of a married girl, they were asked about the circumstances around her decision to marry and decisionmakers involved. This was then followed with questions from the discussion guides.

### Data management and analysis

We audio-recorded all FGDs and interviews and transcribed them into Arabic. The first author checked a sample of the transcripts to ensure accuracy of transcription. Regular debriefings took place with the local team to discuss challenges, emerging themes from the data, and areas requiring further research. Data collectors were encouraged to practice reflexivity by keeping fieldnotes throughout the data collection process and providing thick description of their interactions with participants. They were also asked to answer a set of questions after every interview to help them analyze their role in the research process and reflect upon how they may have implicitly or explicitly influenced research findings.

A codebook was developed with the local team using deductive codes – identified a priori – based on the research objectives. This was coupled with an inductive approach in which we applied initial coding followed by focused coding – as described by Charmaz [41]. During initial coding, a sample of thick, contextually-rich transcripts was selected and scrutinized line by line which allowed for codes to emerge from the data – including in-vivo codes that preserved the participants’ language and terminology. The most useful initial codes were then selected and tested against the data. Coding of the Arabic transcripts in addition to detailed memoing^1^ were done by the first and second authors who are Arabic speakers and who have graduate-level training. Dedoose v 8.2.27 [43] was used to facilitate data management and organization and allowed the first and second author to collaborate from Baltimore and Cairo. In August 2019, the first and second author organized an analysis workshop to review preliminary findings with the in-country data collection team as well as local partners. The workshop helped establish the credibility and accuracy of the findings and provided an external check to the analysis process [44].

### Ethical review

Ethical approval for the research was secured from the Johns Hopkins Bloomberg School of Public Health Institutional Review Board (IRB) [Ref number 9276] and the Egyptian Society for Healthcare Development Research Ethics Committee [Ref number 1/2019]. We received permission to obtain verbal consent from adult participants and married girls ages 16–17 who are considered emancipated minors in this context. An oral assent process was approved for unmarried children 10–17 and married children under age 16. This was coupled with oral permission from their parent/guardian.

## Results

We first present a summary of participants’ views and perceptions around marriage and note the ways in which marriage practices and customs are perceived to have changed in the aftermath of displacement. We then describe sociocultural and economic factors which sustain the practice of child marriage. To this end, we present findings from the participative ranking exercise in which drivers of child marriage were elicited and ranked by participants. We also identify a number of positive changes with implications for family formation dynamics following displacement into Egypt. Finally, we present experiences of married adolescent girls, while drawing attention to the myriad ways in which child marriage impacts the health and overall wellbeing of girls in this context.

### Marriage customs and arrangements

Key informants confirmed that child marriage – followed closely by childbearing – is common among Syrian refugees residing in Egypt. As articulated by a UNHCR protection officer:

“Many Syrian families prefer early marriage because of prevailing cultural norms and fears of being stigmatized by community members. The frequent use of the term *‘anis* [spinster] demonstrates the stigma attached to girls who get married at an older age. What is surprising is that age at which a girl becomes a *‘anis* in this community can be as early as 18-20 years old.” UNHCR protection officer, Cairo

Accelerated by marriage, the transition from childhood to adulthood was generally portrayed as abrupt, without a distinct period of adolescence, and married girls described being prematurely forced into adult social roles. The photo elicitation exercise illustrated the centrality of marriage to the lives of girls. Almost everyone who was shown a picture of a young girl with a child was quick to identify the image with marriage and early childbearing. As put by a married girl when asked about what makes her think the image represents a mother holding her child: “At her age, we all had a child of our own.”

When asked to envision a day in the life of the girl whose image was presented, almost all participants gave formulaic responses, listing household chores like cleaning, cooking, tending to children as the daily routines of married girls. None described work or school-related activities. Participants described strong normative gender roles which assigned women to the domestic sphere, and which prioritized men’s roles as breadwinners and providers for the household.

While marriage did not typically occur within a context of outright coercion by parents, most participants explained that arranged marriages prevailed as the dominant form of marriage. Initiating relationships was perceived as socially unacceptable, and the dwindling pool of eligible suitors due to displacement was said to encourage arranged marriages. Despite stating that they were given space to refuse suitors, dominant gerontocratic and social norms detracted from girls’ autonomy and ability to assert their preferences. This was illustrated in many girls’ accounts in which they described being “nudged” to accept a marriage proposal by their parents whose anxiety about finding a suitable groom instilled an urgency to marry girls as soon as an opportunity presented itself. While most participants noted that mothers wielded considerable influence with respect to decisions around marriage timing and readiness, they still noted that decision-making power rested overwhelmingly with the male figure in the family – most often the father.

Nonetheless, even though child marriage was normalized in pre-war Syria, there were signs of changes in marriage and family formation following displacement. Many girls and their parents explained that norms supporting earlier marriage were beginning to erode, due in part to the displacement to Egypt and exposure to the Egyptian way of life. In fact, when asked about ideal age at marriage, almost all girls stated that they preferred a later marital age – preferring to wait till they turned 18 or after. Notwithstanding heterogeneity in responses about preferred marital age, most parents also agreed that they would rather wait till their girls reached age 18 or 20. Interviews with UNHCR staff further confirmed that child marriage cases are declining among beneficiaries of their programs, despite an increasing caseload of other GBV types.

### Drivers of child marriage

The participative ranking exercise shed light on several drivers of child marriage, many of which were subsequently mentioned during the discussions. The most commonly cited drivers across groups were 1) cultural norms and traditions; 2) groom availability and the concept of *qisma w naseeb* which translates into share and portion but is understood as destiny; 3) economic burden stemming from displacement; 4) education; and 5) protection concerns. Conspicuously absent in participants’ responses was the role religion plays in motivating marriage. Unless directly probed about religion, participants rarely mentioned it as a driver of child marriage and when asked, only a few cited religious prescriptions that encourage marriage as soon as a suitor with ‘good character’ is identified:

“According to the prophet: ‘When someone whose religion and character you are pleased with proposes to (someone under the care) of one of you, then marry him. If you do not do so, then there will be turmoil (*fitnah*) in the land and abounding discord (f*asad*)’."– (Syrian father, FGD, Giza )

#### Culture and traditions

For some participants, child marriage was now a relic of the past but for others, longstanding traditions and customs – which predate the influx of Syrians into Egypt – set child marriage as a social necessity. Those customs were viewed as especially dominant among families from rural areas in Syria. Indeed, several participants stated that tradition was the foremost driver of child marriage, trumping economic considerations, and that it was “in [their] nature” to marry off girls early. As articulated by one participant:

“It is in our cultural traditions in Aleppo, if a girl reaches that age, she must get married, if she waits until she is maybe 20, she is no longer desired.” (Syrian mother, FGD, Qalyubia)

“It is entirely tradition that motivates some families to marry their daughters – not poverty. Where we are from, it is the norm. But many people have changed and have started to learn from the mistakes they made” (Syrian mother, FGD, Damietta)

The concept of *qisma w naseeb* – which translates into share and portion – permeated parents’ accounts. The concept is used to denote fate and predestination in matters of marriage, implying that every person is awarded with a share of gifts from god, and so many participants were concerned that forgoing their share would guarantee ‘spinsterhood’ and bad luck. Several parents explained that they had no intention to marry off their daughters, until they were approached by a suitor that they “could not refuse.” However, this quote by a Syrian mother identifies the cultural tendency to revert to *naseeb* as a pretext to shift blame:

“We have a big problem in Syrian culture which is that we do things and we blame it on fate. [Everyone in the FGD nods in agreement]. I personally married off my daughter at age 15. She was approached by a good suitor and I manipulated her father into agreeing. You know us women we have considerable impact on our husbands. If I want something, I will nag and nag and after I get my way, I will just say its *qisma w naseeb*” (Mother, FGD, Qalyubia)

#### Gender norms

While none of the participants identified entrenched gender norms as a driver of child marriage, it was very apparent from interviews that normative gender roles and values constituted an underlying contextual driver of child marriage. Parents discerned child marriage as a way to “control,” “mold,” and “shape” girls into being obedient wives. Marriage was also viewed as a means to protect girls’ sexuality, with many participants invoking the concept of *sutra* which refers to “preserving girls’ reputation and family honor.” Mothers also repeatedly cited concerns that girls who join the labor force and postpone marriage become recalcitrant and difficult to control and are thereby perceived as less marriageable.

“I have a neighbor who has a 20-year-old daughter who gets approached by so many suitors, but she does not like any of them. She has now reached an age where she wants other things, she wants to work, she wants to go out and have a life. Her mother would cry to me and say I wish I had married her off early because now she has all these conditions and stipulations.” (Syrian mother, FGD, Qalyubia)

#### Economic insecurity

For some families, entrenched gender norms interacted with economic insecurity – triggered by the displacement experience – to perpetuate the practice of child marriage. While a few participants mentioned *mahr* or bride price – a customary payment from the groom to the bride’s family which was now defined in US dollars due to displacement – as a driver of child marriage, many confirmed that the prospect of shifting the financial burden of a girl onto her groom was more important than bride price.

“This family had too many children, both girls and boys, and so the father was unable to support his girls. When a suitor presented himself, and was financially capable, the father decided to marry off his daughter. This way he could better support his other children; it reduced the burden.” (Unmarried girl, Giza, IDI)

#### Schooling-related drivers

Of great salience was the link between child marriage and access to education which not only emerged in the participatory exercise but was woven into most participants’ accounts. Parents expressed a preference for marrying off girls who were less “inclined” towards education. Those who were not excelling in school or who did not show interest in education were better off getting married, whereas girls who showed a serious desire for education were retained in school and their marriage was postponed. As articulated by a father interviewed in an FGD in Giza “education is not more important than marriage. If a girl does not lean towards education, then it is best that she is married.” Even girls echoed this sentiment; an unmarried girl from Damietta explained in an IDI: “When a girl is not interested in studying and prefers staying home. If she is not focusing on studying, or not doing well in this regard, her parents will decide that she is better off getting married.” Perhaps not surprisingly, this opinion seemed to apply only to daughters and the same logic did not seem to extend to sons.

Against a backdrop of displacement and instability, many participants reported challenges with school enrollment. This resulted in some parents taking their girls out of school and resorting to child marriage due to the lack of viable alternatives. As explained by a father who escaped violence in Ghouta, Syria:

“I spent over a month and a half in a basement in Ghouta. Of course, my children were not in school, they could barely eat and drink. We didn’t move for a month and a half from that basement. Then I came here, arriving three and a half months after the school year started. So I couldn’t enroll my children in school.” (Father, FGD, Giza)

Another explained that the lengthy process of regularizing his residence and that of his children complicated school access:

“I came here three years ago, tried to register my daughter in school, and they said we cannot do that until you have obtained a residence permit. I came in the middle of the school year, so obviously by the time I got residence it took another six months. I still remember the day I got the permit. I went from Tahrir to the educational administration to register the kids, they told me it’s too late, the school year is halfway through and I should wait until next year. The new year came, and I went to enroll them, but was told they would have to repeat grades. They said my son would have to repeat first grade despite completing it in Syria.” (Father, FGD, Giza)

Interviews with key informants confirmed challenges faced by refugees in accessing education. While the government grants Syrian refugees residence permits, they are valid for six months only and their renewal is complicated. Without residence permits, parents are unable to register their children in schools.

Protection concerns converged with the lack of quality education to heighten the appeal of child marriage for a subset of participants, especially those who could not afford private schools. Parents repeatedly decried the quality of public schools in Egypt. Many participants protested the over-crowdedness of Egyptian public schools, cited challenges their children had with understanding the Egyptian dialect and complained that their children experienced bullying for being Syrian. At the same time, many parents articulated fears that their daughters would be subject to sexual violence on the way to and inside school, especially since unlike schools in Syria, many schools in Egypt were mixed gender. Fears of sexual violence were most frequently cited by participants living in Qalyubia. This could be in part explained by the fact that the governorate hosted many factories which were staffed by single male workers migrating from neighboring governorates who were not known to the residents of the city.

As articulated by a mother concerned about her daughter’s safety:

“My daughter loves school, but I want her to drop out. I am very scared of the company she keeps. If I were in Syria I would not have felt the same way but here things are different. I do not feel safe about her going out alone. I would rather she get married so I feel safe.” (Mother, FGD, Qalyubia)

Another girl in Qalyubia described why her parents made her drop out of school:

“I was in the eighth grade and I was wearing the niqab. Because, the schools here are mixed, I had a hard time, because of the boys. One time, a boy followed me home and jumped on top of me. My brother interfered and protected me, but since then I have hated school” (Married girl, IDI, Qalyubia)

### Positive impacts that participants associated with displacement

For many participants, however, displacement to Egypt relaxed social rules that had curtailed girls’ mobility and education attainment. In fact, many girls interviewed were well-versed in rights-based language and very assertive about their plans for their future, confirming that they were intent on pursuing an education and delaying marriage. This empowerment was coupled with a readiness on the part of many parents to keep girls in school because it was deemed acceptable in the host community for girls to pursue an education. As put by one participant:

“I would have never imagined that *baba* would let me study; he used to reject the idea of me leaving the house, but since we came here, he let me leave the house to go to school. He agreed because he saw that people in Egypt cared about education and allowed it, so he let me.” (Unmarried girl, IDI, Damietta)

Fueled by the circumstances surrounding their displacement, many parents emphasized the importance of education in what they described as “dark times.” As explained by one father in an FGD in Giza, “Right now girls ought to be educated not married off.” Another participant explained:

“After experiencing displacement, girls are more aware and more interested in pursuing an education. Many girls who are pursued by a potential suitor, refuse to be married and demand to continue their education. This was the case after we settled. When we first came to Egypt, people were opting to marry off their daughters, but now things have changed.” (Mother, FGD, Damietta)

Key informants also confirmed that Syrian refugees were becoming increasingly influenced by their Egyptian host community and as such, women were starting to seek employment opportunities, which was uncommon for women in pre-war Syria. Humanitarian actors interviewed in the study explained that the inclusion of livelihood and economic empowerment components in their programming – which have intensively targeted Syrian refugees – may have incentivized women to seek employment, thereby challenging long-established gender norms that discourage work among women.

Displacement was seen as affecting child marriage in other ways as well. Shifts in family structure due to displacement – exemplified by the separation of families and the reduced role of grandparents as decision-makers – were perceived as disrupting traditions which previously perpetuated child marriage. Many participants explained that it was increasingly difficult to find grooms for their daughters – given their preference to marry their daughters to men from the same city or village back in Syria. They also reported declines in cousin marriages which reduced child marriage for some girls – albeit in some cases it was noted that the dearth in grooms expedited marriage when a proper suitor was identified.

### Consequences of child marriage

In examining consequences of child marriage, we primarily relied on accounts of married adolescent girls, but complemented that analysis with insights generated from interviews with key informants, parents and unmarried girls. We present findings relevant to four themes which emerged as most salient: 1) sexual and reproductive health impacts; 2) other forms of GBV; 3) impacts on education and livelihood; and 4) marriage and birth registration.

#### Sexual and reproductive health impacts

Key informants emphasized the prominence of cultural norms that give primacy to women’s roles as mothers and that promote childbearing. Beliefs that a girl’s worth is intimately tied to her fertility prompted married adolescent girls to commence childbearing as soon as they got married. Most married girls interviewed in the study had their first child soon after marriage and the majority stated that they had difficult pregnancies and experienced complications. Very prevalent was the belief among married girls and mothers that using modern contraceptives causes infertility among nulliparous women and thus rarely did adolescent girls report using contraception. When they did, it was mostly after the first birth, and even then, they reported using withdrawal and periodic abstinence in lieu of modern contraceptives.

Some health providers also encouraged girls not to use hormonal or “invasive” methods. One participant described the reluctance of her health provider to insert an IUD:

“Here they won’t let you use an IUD until you have had your first boy. I struggled a lot and my doctor refused to insert the IUD because she said it could cause infertility. She said I should instead use natural methods or if I must, then I should use oral contraception. I chose the natural methods.” (Married Syrian girl, IDI, Qalyubia)

Another OB/GYN explained:

“I prefer that a young girl with no history of childbirth use the pill and not the IUD, because the IUD can cause bleeding. Her body is still too young.” (OB/GYN, KII, Damietta)

#### Child marriage and other forms of gender-based violence

The impact of child marriage on gender-based violence was not clear cut. A few participants stated that girls married at a young age were more likely to be subjected to violence due to the fact they are being “molded” by their husbands, and thus need to be “disciplined”. This was said to reduce with time as married girls got older. Yet while wife beating was justified as “discipline” only by a minority of participants, non-physical violence was much more widely accepted.

Female genital mutilation (FGM) – a practice prevalent in Egypt which is rumored to have gained popularity among Syrian refugees since their arrival in Egypt – was vehemently denounced by all participants. None reported undergoing FGM or hearing of anyone who did. Humanitarian actors and community leaders also confirmed that they had never come across such cases. Asked what he would do if a prospective suitor requested that his daughter undergo FGM before marriage, a Syrian father in an FGD in Giza answered: “I would rip his head out.” Another stated that the practice was never done in Syria and that “we don’t have it, didn’t know it and don’t want to know it.”

#### Impacts on education and livelihood

Many girls who were married before the age of 18 reported dropping out of school soon after marriage. They also stated that they were not allowed to work outside of the household. Dictated by the husband, sometimes in consultation with the father-in-law, restrictions on women’s education, mobility and employment were justified by prevailing beliefs about a woman’s responsibility to attend to household chores, look after the husband and children, and abide by cultural norms that prescribe a wife’s adherence to the private sphere. Limitations imposed on physical mobility were often described as “attempts to ensure women’s safety.” Participants frequently depicted Egyptians as espousing more open and liberal social norms which allow interaction between men and women on a daily basis. In contrast, Syrian husbands believe that public interaction is reserved for men and due to jealousy and safety concerns prefer to curtail women’s mobility and social networks.

As put by a married girl residing in Damietta when asked about the possibility of her husband accepting her entry into the workforce, “I assume if the place of employment is mixed-sex or has many male employees, my husband will refuse sending me to work as he gets jealous.”

In multiple interviews, women stated that it is culturally frowned upon to seek employment as it is the sole responsibility of the man to provide for his family. In desiring to work, a woman would be implying that a man has failed to honor his duties and responsibilities as a breadwinner. A quote from a married girl from Giza further illustrates this sentiment: “Our men believe that sending off your wife to work is a shameful matter. They would say ‘I’m a man. Am I not enough for you and the family?’”

While there were some participants who said they knew of girls who continued their education after marriage, it was indicated that this was uncommon and that girls would run the risk of getting divorced if they choose to do so. In some cases, married girls explained that their spouses had falsely promised to allow them to complete their education or work after marriage, but then refused to do so. An unmarried girl in Al Qalyubia recounted the story of her sister:

“My sister got married to a man who promised my father that she’ll continue her education after marriage. A day after the wedding, my sister brought up the topic and he responded by saying that his agreement with my father was otherwise and that he never agreed to her completing her education. Although he lied, she still remained in her marriage because it is considered shameful to get divorced.” (Unmarried girl, FGD, Qalyubia)

#### Marriage and birth registration

Within the context of displacement, marriage registration of girl brides is viewed as challenging. As indicated by most participants who got married under the age of 18 in Egypt, they circumvented the Egyptian legal system by having their marriages officiated by a sheikh/imam (commonly referred to as *Katb Kitab*). Given that marriage under age 18 is banned in Egypt, some participants reported that sheikhs/imams asked for bribes to officiate their marriages. In order to officially register the marriage, most married girls noted that it needed to be first recognized by a court of law in Syria. There, marriage is allowed under age 18 by judicial exception. As noted by a key informant:

“Because the law is permissive, the practice of child marriage has flourished in Syria. Allowing exceptions to laws on minimum age at marriage is very problematic. It happens often that judicial exception is sought by a family, and the judge simply needs to ask a few questions to determine a girl’s readiness for marriage.” (Lawyer, KII)

Since both husband and wife resided in Egypt, participants reported registering their marriage through the power of attorney, which was usually given to a father, a grandfather or an uncle who resides in Syria. For a few participants, their husbands went back to Syria to register the marriage themselves. The husbands would eventually send their wives the marriage contracts. However, it was highlighted that this was a risky endeavor because in some cases, the husband neither sent back the contract nor returned.

If the wife became pregnant before registering her marriage, as was the case with some participants, birth registration was described as difficult but not impossible. Most participants are able to register the birth in Syria and the birth certification was acknowledged in Egypt. However, this process took considerable time and required that the father be present. In the absence of a father, birth registration is not possible, and women are at heightened vulnerability because they cannot obtain a birth certificate. A married girl in Damietta explained:

“I had a hard time trying to register the birth of my child. No one from his father’s side is here in Egypt and his father is currently serving in the Syrian army. We can’t bring him here during his military service. But the authorities finally agreed to having the father’s cousin to fill in his place and attest to the kinship between my son and my husband. We are currently processing the papers to receive the birth certificate.” (Married girl, IDI, Damietta)

There were widespread misconceptions among girls and mothers about access to public health services in the absence of a birth certificate. Health providers confirmed that vaccination and other child health services were fully provided even if the mother was unable to furnish a birth certificate. Instead, a birth notification – given to all women who deliver in hospitals in Egypt – was sufficient to enable access to health services. Unlike health services however, registering children in schools was impossible without the child having a birth certificate. Women who had been unable to secure birth certification for their children faced serious challenges in enrolling their children in school.

## Discussion

Our study sheds light on the marriage practices of Syrian refugees residing in mostly urban areas in Egypt and examines drivers and consequences of child marriage in this context of protracted displacement. An already prevalent phenomenon in pre-war Syria, child marriage was sustained after the influx into Egypt by pre-existing cultural traditions and gender norms that prioritize the role of girls as wives and mothers. However, in addition to pre-existing drivers, we found that challenges arising from displacement such as disruptions to girls’ education, protection concerns, and livelihood insecurity exacerbated the vulnerability of many families, positioning marriage as a coping mechanism. These challenges however differed in magnitude across governorates, with protection concerns for example most salient in Qalyubia, perhaps due to the highly industrialized nature of the governorate, but less relevant in other governorates. Our findings align with other literature on drivers of child marriage among Syrian refugees in the region [28, 29, 45, 46].

Notwithstanding, we find some evidence that while challenges associated with displacement may have hastened marriage for many girls, for others, factors associated with displacement may have contributed to postponement of marriage. Although the lack of quantitative data precludes conclusions about changes in prevalence of child marriage, our qualitative data indicates that drivers of child marriage in this context of displacement are multi-faceted and varied. In this case, for some groups, their protracted presence in urban areas in Egypt may have facilitated the erosion of social rules and expectations which favored child marriage. Norms around gender roles appear to be changing, with more tolerance for girls completing school and engaging in livelihood activities in part due to the normalization of girls’ education and employment in the host community as well as the shift in opportunities that may be now more readily accessible to girls. Secondly, changes in family structure and separation from older family members – such as grandparents – may have disrupted traditions that previously perpetuated child marriage. Combined with the unavailability of grooms due to forced migration, difficulties in securing a suitable spouse may have led to marriage postponement for some girls. Additional insight into how child marriage programs targeting refugees and other crisis-affected populations have been effective in changing attitudes and marriage practices, as well as opportunities for girls and families, merits further examination.

That displacement may contribute to reductions in child marriage has been quantitatively shown in other contexts [46–48]. In the aftermath of the Lebanese civil war, “marriage squeeze,” the phenomenon whereby an imbalance takes place between numbers of males and females in prime marriage ages, was documented [48]. Due to the deficit in males in Lebanon arising from excess mortality and emigration among males as well as economic woes, delays in age at first marriage were observed. Similarly, increases in proportions of never-married women in the age group 10–19 were noted during the second intifada in Palestine – likely due to economic difficulties [49]. The finding that in this setting, displacement may have engendered different responses with respect to marriage warrants further empirical investigation.

Consistent with other studies, our findings highlight the interplay between displacement, education, and child marriage. We describe several pathways linking education to child marriage which lend themselves to interventions that promote school retention. Streamlining the process by which refugees regularize and renew their residency in Egypt has the potential to remove barriers to education. Currently, the government of Egypt grants refugees six-month residence permits and their renewal is a cumbersome process that is only possible from Cairo. Advocacy by humanitarian actors to prolong the duration of residence permits and to decentralize the process would not only improve refugees’ sense of stability and security but can facilitate school enrollment and retention for refugee children. We documented other barriers to education such as fears of sexual harassment, over-crowdedness and bullying in schools. Addressing these challenges can improve school access and quality, and consequently school retention, possibly leading to changes in marriage practices in this population.

Other ways through which education was found to impact child marriage are more complex and intractable. Norms that devalue education of girls – especially those who are deemed “disinclined” towards education – and that prohibit girls from joining the workforce were evident. These norms are perpetuated by the belief that girls who postpone marriage are more likely to become defiant and harder to control and are compounded by the fact that because women are not usually allowed to work, there was not much perceived benefit to investment in education. While we offer evidence that norms may be slowly changing and that caregivers are becoming more cognizant of the value of girls’ education, harmful social norms remain.

Furthermore, our study findings do not support claims that FGM has gained popularity among Syrian refugees. All study participants, without exception, strongly rejected the idea of cutting girls and none espoused the belief that FGM makes girls more marriageable. This is not surprising given that most Syrian refugees resided in urban areas where FGM is less commonly practiced among the Egyptian host community [50]. That and the finding that Syrian families preferred to marry off their daughters to Syrian men may have prevented adoption of the practice. Whether the small groups of Syrian refugees who reside in rural areas, where the practice is more widespread, have adopted the practice is unknown and requires further investigation.

Our study also illustrates the range of social, health and economic impacts of child marriage among this group of refugee girls. As documented in several other settings [9, 46, 51–53], early childbearing and reluctance to use contraceptive methods until after the first birth were almost universal in the study sample. Fueled in part by pronatalist norms and taboos against contraception, the low levels of contraceptive use are compounded by provider biases against certain methods. Seeing as long-acting reversible contraceptives (LARC) like the IUD and implant are highly effective in delaying pregnancy and are safe to use irrespective of age [54], efforts to dispel provider misconceptions regarding the appropriateness of LARCs and other hormonal methods for adolescent girls are needed. These should be coupled with family planning interventions targeted at married adolescent girls and their spouses.

Because child marriage is illegal in Egypt, marriage registration emerged as a factor potentially exacerbating girls’ vulnerability. Most families resorted to religious marriages, officiated by sheikhs and imams, and many did not pursue legal registration, which had implications not only for the women but for their children’s birth certification and access to schooling. This resonates with findings from other studies conducted with Syrian refugees in Lebanon in which marriage and birth registration emerged as salient issues [28, 30]. Discrepancies between laws in Syria and Egypt however created loopholes which allowed some refugees to register marriages in Syria. To the extent that they were able to do so, Syrian women who gave birth before turning 18 were eventually able to obtain birth certificates for their children. This served to protect them since their children would have otherwise been unable to access some important services. Unlike their Syrian counterparts, however, Egyptian girls are unable to register marriages prior to turning 18 and are in comparison more vulnerable along with their children. Drawing attention to this issue, a recent study conducted on child marriage in the Middle East region identified birth certification as a major challenge for child brides, and advocated for granting families access to birth certification irrespective of the status of their parents’ marriage [20].

Our study has several limitations that must be noted. For one, we interviewed participants in three governorates in Egypt and thus the extent to which our findings are transferable to refugees residing in other areas is uncertain. Despite that external generalizability is not necessarily considered a goal of qualitative studies [55], we have no reason to believe that our findings do not apply to Syrian refugees residing in other urban areas in Egypt. Moreover, given the sensitive nature of our inquiry and the fact that many of the participants were users of UNFPA and UNICEF services, we are unable to dismiss the possibility of social desirability bias. However, the risk of bias was mitigated by efforts to recruit new service users who may have not yet been exposed to messages against child marriage. Still, there are many programs targeting Syrian refugees in the study sites, and we cannot claim that our participants were not already sensitized to the dangers of child marriage.

Limitations notwithstanding, our study has several strengths. We purposively sampled and interviewed a large number of married and unmarried girls, father and mothers to gain a comprehensive understanding of child marriage drivers and consequences. Additionally, all data collection and analysis were carried out by local researchers, who are well acquainted with the context and language. Peer debriefing and review took place to validate the findings of the study. To our knowledge, this is the first study that provides an in-depth analysis of child marriage practices of Syrian refugees residing in Egypt.

## Conclusions

Our study paints a textured and nuanced picture of child marriage among Syrian refugees living in Egypt. Despite uncovering similar insights to studies conducted with Syrian refugees in other contexts, our analysis adds to the knowledge base on drivers of child marriage in displacement settings. Our findings have the potential to inform targeted, focused, and contextualized interventions that not only prevent child marriage in this setting but also mitigate its impacts. Coupling social and behavioral change communication focused on families (including boys and men) with access to quality services - education, health, GBV, and social protection - for adolescent girls, young women, and their children can transform norms, opportunities, and eventually marriage practices.

## Supplementary Information

**Additional file 1.** Focus group discussion guides.

**Additional file 2.** In-depth interview guides.

**Additional file 3.** Key-informant interview guides.

## Data Availability

The dataset supporting the conclusions of this article is available on request from the corresponding author.

## Abbreviations

FGM: Female genital mutilation
FGD: Focus group discussion
IDI: In-depth interview
IRB: Institutional Review Board
KII: Key Informant Interview
LARC: Long-acting reversible contraceptives
PRM: Participative ranking methodology
PRA: Participatory rapid appraisal

## Footnotes

1 Memoing is “the act of recording reflective notes about what the researcher is learning from the data” [42]
