# Supplementary material for: Drivers and consequences of child marriage in a context of protracted displacement: a qualitative study among Syrian refugees in Egypt
Source: BMC Public Health. 2021 Apr 7;21:674. doi: 10.1186/s12889-021-10718-8 (PMC8028254; doi:10.1186/s12889-021-10718-8)
Supplement: Supplementary file 1 — Additional file 1. Focus group discussion guides. [file 12889_2021_10718_MOESM1_ESM.docx]

**FGD Guide for Fathers**

Introduction

I would like us to start with an activity in which we get up and work together. All of you have girls under the age of 18. I would like you to think of the main reasons why fathers may want their girls to get married at this age in your community in Egypt.

*Go ahead and write down some ideas you have on the sticky notes handed to you. After you are done, please stick the notes on this blank whiteboard. Let’s discuss some of the things you wrote down*.

Pick two/three things to discuss that are relevant to the research.

*Now, I will ask you to agree with each other on the order of these reasons. Please order the sticky notes in a straight line. The biggest reasons should go at the top of the line, and the less important reasons at the other end of the line.*

Participants should discuss the ordering of the sticky notes and the notetaker should carefully record why they have chosen to sort the items in this order.

Now, I would like to move on to asking you some more questions about being the father of a young girl in this community.

1. What are the biggest challenges you face as a father of a young girl in this community today?

(Probe: Protection concerns, financial problems, livelihood/occupation, social status)

1. When thinking about your role as a father, how are you expected to ensure that your daughter has the best future she could have?
2. How has displacement changed how you think about your role as a father?

Topic 2: Perceptions and traditions around marriage and relationships

These next questions are about marriage.

1. When do you think is the appropriate age for your daughter to get married?
   1. Is this the same age as before you came to Egypt?
2. What do you think makes a young girl ready to get married?
   1. Age
   2. Physical features
   3. Family
   4. Religious considerations
   5. Biological considerations
   6. Culture? Traditions? Origin?
   7. Household skills
3. What are the characteristics that you would look for in a husband for your daughter?
   1. How have these characteristics changed since you came to Egypt?
4. What are the necessary steps you would have to go through if you wanted your daughter to get married? (Probe: Who would have to be consulted? What exchanges (mahr) or ceremonies would have to take place?)

Topic 3: Drivers of child marriage

1. What are some of the advantages of your daughter getting married?
   - 1. What are economic advantages of marrying early?
     2. What are some social advantages (social status, education, social life/friends)?
     3. What are health-related advantages?
     4. Does marriage offer protection to girls? How so?
2. What are some disadvantages of your daughter getting married?
   - 1. What are economic advantages of marrying early?
     2. What are some social advantages (social status, education, social life/friends)?
     3. What are health-related advantages?
     4. Does marriage offer protection to girls? How so?
   1. How are these advantages and disadvantages related to being a refugee in Egypt?

Topic 5: Local traditions and harmful practices

1. Among Egyptians, FGM is sometimes a prerequisite to marriage. How has the perception of FGM changed among Syrians since you came to Egypt?
   1. What would you do if a prospective husband asks that your daughter be cut?
2. Do you know/ have you heard of any Syrians who have practiced FGM? If so, tell us about their experience.

Probe:

1. What influenced the decision?
2. Who where when
3. At what age
4. What are consequences (legal/ marriage dynamics/ health consequences + complications/
5. How does this change how she is perceived within her community and the Egyptian community.

Topic 6: Access to services

Now I would like to ask you some questions about life in this area.

1. What services do you think are necessary for your daughter?

Probe

Education

Health

Livelihood training

Employment training

- 1. How do you support her accessing these services?

1. What obstacles do you face in supporting these services?
   1. What are some ways that you have tried to cope with these obstacles?

**FGD Guide for Mothers**

Introduction

I would like us to start with an activity in which we get up and work together. All of you have girls under the age of 18. I would like you to think of the main reasons why families may want their girls to get married at this age in your community in Egypt.

Go ahead and write down some ideas you have on the sticky notes handed to you. After you are done, please stick the notes on this blank whiteboard. Let’s discuss some of the things you wrote down.

Pick two/three things to discuss that are relevant to the research.

Now, I will ask you to agree with each other on the order of these reasons. Please order the sticky notes in a straight line. The biggest reasons should go at the top of the line, and the less important reasons at the other end of the line.

Participants should discuss the ordering of the sticky notes and the notetaker should carefully record why they have chosen to sort the items in this order.

Topic 1: Perceptions and traditions around marriage and relationships

- 1. **Describe to us the norms/practices related to marriage and relationships in your communities in Egypt**

Probe:

- - 1. How did girls learn about relationships in Syria?
    2. How did girls meet their prospective husbands?
    3. Who decided whether a girl was ready to get married?
    4. Who facilitates the marriage process?
    5. What factors were considered before a girl was deemed ready for marriage?
    6. How common was marriage of cousins? Why/why not?
    7. How was bride price (mahr) negotiated?
    8. How long did the marriage process typically take?
    9. How often do men take a second wife?
  1. **In what ways have marriage practices and traditions changed since the arrival of Syrian refugees?**

Probe

- - 1. How has competitiveness changed?
    2. What changes took place with regards to financial costs of marriage and mahr?
    3. Did the profile of wives change? How? (nationality / age / socioeconomic status/ cousin?)
  1. **What does society think about the age at which girls should marry? Boys? Do you agree? Why/Why not?**
  2. **How do you think age at marriage changed since you were displaced to Egypt?**

Probe

- - 1. How did age at marriage change for girls?
    2. How did age at marriage change for boys?
  1. **If daughter is unmarried: Suppose your husband was approached by a man for your daughter’s hand. How would you and your husband react? Why?**

Probe

- - 1. Would you both have the same reaction? How so?
    2. Who would ultimately make the decision?
    3. What factors would impact whoever is making the decision? (spouse’s age/wealth/nationality)
    4. What role would the girl’s views and preferences play?

Topic 2: Drivers of child marriage

- 1. **In your opinion, what are some advantages to marrying girls at an early age?**

Probe

- - 1. What are economic advantages of marrying early?
    2. What are some social advantages (social status, education, social life/friends)?
    3. What are health-related advantages?
    4. Does marriage offer protection to girls? How so?
  1. **What are some of the disadvantages to marrying girls at an early age?**

Probe

- - 1. What are economic disadvantages of marrying early?
    2. What are examples of social disadvantage (how does CM impact a girls education, her ability to work)?
    3. Health-related disadvantages?
  1. **What are the challenges faced by girls who are married at a young age?**

Probe

- 1. Tell us about the marriage registration process you go through in Egypt for young girls
  2. Do girls who marry at a young age face problems accessing health services? Tell us about some of those problems
  3. In some cases married couples fight or show anger toward each other. Oftentimes this can mean physical harm. Do you see this practice happening among young married couples?
  4. How do you think displacement impacts the challenges you mentioned? Have some of these challenges changed since you came to Egypt?
  5. **What are the main reasons why people in your community choose to marry their girls at an early age?**

Topic 3: Experience of child marriage

**3.1 Do you know of someone in your community who married at an early age. Tell us about her experience.**

Probe

- - 1. How did she meet her husband?
    2. What role did her parents and community play? Who were the decisionmakers involved?
    3. What factors impacted whoever made the decision? (spouse’s age/wealth/nationality)
    4. Why was the decision made to marry her at such a young age?

**3.2 In your opinion, what changes occurred in her life as a result of the decision to marry her at a young age?**

Probe

- - 1. How did marriage impact her education?
    2. How did marriage impact her ability to work?
    3. How did marriage impact her social networks? Social standing?
    4. How did marriage impact her emotional wellbeing?
  1. **When you think about your daughter’s future, what’s the best future you imagine for her?**

Topic 4: Local traditions and harmful practices

- 1. **How does the local context in Egypt affect marriage practices and norms in your community?**
     1. How does local context affect who a girl gets married to?
     2. How does local context affect bride price?
     3. Many Egyptian men will require that a girl undergo FGM before she is married? If an Egyptian man shows interest in your daughter but stipulates that she undergo FGM, what would you do?
     4. Describe to us an instance where you witnessed/heard of an Egyptian man/family asking that a girl undergo FGM as a prerequisite to marriage. How did the family react?
        1. Probe if yes
        2. What age was the girl/ Where did it happen/ Who did it/ How did it influence marriage dynamics/ What are some consequences of that action?

Topic 5: Access to services

Now I would like to ask you some questions about life in this area.

- 1. What services do you think are necessary for your daughter?

Probe

Education

Health

Livelihood training

Employment training

- 1. How do you support her accessing these services?
  2. What obstacles do you face in supporting these services?
  3. What are some ways that you have tried to cope with these obstacles?

**FGD guide for Married Girls (under the age of 18)**

Topic 1: Introduction

Show two drawings of married and unmarried girls. Start by introducing the first picture and ask participants to describe what they see. Show a picture that depicts an unmarried girl. Ask participants why they think the girl is unmarried. Can they describe her daily activities? When do you think this girl will get married? Who will be involved in her decision to get married. Present a picture that depicts a married girl. Ask participants about why this girl is married, who had an influence on her, what they think her daily activities are, what environment she grew up in, what her current environment now looks like. Ask about the circumstances around her decision to marry. Was it her decision or was it made by others? If so, who?

**Introduce the topic of the discussion: marriage of young girls.**

Topic 2: Perceptions around marriage and relationships

- 1. **We are interested in understanding how young girls learn about relationships and what their primary sources of information are. Can you tell us how young girls in your community learn about relationships?**

Probe

Who are the sources of information on marriage and relationships?

Mothers?

Female relatives?

Peers?

Teachers?

Internet?

How have sources of information on marriage changed after you came to Egypt?

- 1. **In your opinion, what does society perceive as a good age for a girl to get married? Do you agree with this perception? Why/why not?**

How has this changed since you came to Egypt?

- 1. **How about for boys, what do you think society perceives is a good age for boys to get married? Why this age? Do you agree? Why/Why not?**

How has this changed since you came to Egypt?

- 1. **Some parents may prefer to marry their girls off at an early age. Why do you think parents may want their girls to marry young?**

Probe

What are economic reasons if any?

Are there any protection concerns that might be driving their decision? What kinds of concerns?

What role do religious beliefs play in the decision-making process?

Do parents acquire a different social status if their daughter is married? Please explain

Topic 2: Marriage arrangements and decision-making

- 1. **In your community, who makes the decision about whether a girl gets married or not? What role do girls play in the decision-making process?**

**Probe**

What role do parents play in this decision?

What role do grandparents play in this decision?

What role do other members of the community play in this decision?

Who facilitates the process of marriage?

How are these decisions made?

1. **What qualities are desired in a prospective husband? (nationality / age / socioeconomic status/ cousin?)**

What is the perception of marriage to men who already have a wife?

How has this changed since you came to Egypt?

- 1. **A lot of families require husbands to pay a bride price. Talk to us about bride price and how bride price is negotiated in your community**

**Probe**

Is bride price given at marriage in your community?

Was it given back when you were in Syria?

Is it the same as it used to be?

Who decides bride price?

How do you feel about bride price?

How is the mahr used? How is this different from when you lived in Syria?

Are there any other exchanges that take place during the marriage negotiation process?

Topic 3: Experiences of child marriage

- 1. **We are interested in learning about the daily activities of girls your age who are married. Tells us about a typical day for a girl like you who is married and has similar responsibilities.**
  2. **In your opinion, what changes – good or bad – occurred in your life as a result of getting married?**

**Probe**

Education

Work opportunities

Social consequences

Emotional consequences

- 1. **Suppose you had a girl of your own and you and your husband were considering marrying her off. What are some advantages from marrying her at an early age?**

**Probe**

What are economic advantages of marrying early?

Social advantages (social status/ freedom from family/ education/ social life/friends)

Health-related advantages?

Protection?

Religious duty?

- 1. **What are some of the disadvantages that arise from marrying her off at an early age?**

**Probe**

What are economic disadvantages of marrying early?

Social disadvantage (how does CM impact a girls education, her ability to work)

Health-related disadvantages?

- 1. **What are the challenges faced by girls who are married at a young age?**

**Probe**

Is marriage registration a challenge to girls who marry early? How so?

Do girls who marry at a young age face problems accessing health services?

Are young girls more likely to experience IPV?

How do you think displacement impacts the challenges you mentioned? Have some of these challenges changed since you came to Egypt?

Topic 4: Childbearing

**4.1 After a girl gets married, she is expected to have children of her own. What do you think is a good age for a girl to have children? Why?**

**4.2 How has displacement changed the age at which girls give birth in this community? Why?**

**4.5 What methods do women use when they want to wait before having a child or when they don’t want to have another child? Do you use any such methods? Why/why not?**

Topic 5: Local context and harmful practices

**5.3 Many Egyptians practice female genital mutilation (FGM)? Are you familiar with this phenomenon? Can you tell us what it is? What are your views about it?**

**5.4 FGM can be viewed as a way to make girls more marriageable in Egypt. Have you heard of any Syrian girls who were asked to do this to get married? What do you think about this?**

**Probe**

How have girls in your community responded to such requests?

How have parents responded to such requests?

Conclusion

**6. When you think about the future of your daughter, what’s the best future you imagine for her?**

**FGD Guide for Unmarried Girls (under the age of 18)**

Section 1: Introduction

Show two drawings of married and unmarried girls. Start by introducing the first picture and ask participants to describe what they see. Show a picture that depicts an unmarried girl. Ask participants why they think the girl is unmarried. Can they describe her daily activities? When do you think this girl will get married? Who will be involved in her decision to get married. Present a picture that depicts a married girl. Ask participants about why this girl is married, who had an influence on her, what they think her daily activities are, what environment she grew up in, what her current environment now looks like. Ask about the circumstances around her decision to marry. Was it her decision or was it made by others? If so, who?

**Introduce the topic of the discussion: marriage of young girls.**

Section 2: Perceptions around marriage and relationships

- 1. **We are interested in understanding how young girls learn about marriage and what their primary sources of information are. Can you tell us how young girls in your community learn about relationships? And how they meet their prospective husbands?**

Probe

Who are the sources of information on marriage and relationships?

Mothers?

Female relatives?

Friends?

Teachers?

How have sources of information on marriage changed after you came to Egypt?

- 1. **In your opinion, what does society perceive as a good age for a girl to get married? Do you agree with this perception? Why/why not?**
  2. **How about for boys, what do you think society perceives is a good age for boys to be married? Why this age? Do you agree? Why/Why not?**
  3. **In your community, what is a typical age at which girls get married? What is the typical age of their husbands?**

Probe

Do you know what the legal age for marriage is (for girls/boys)? What do you think about this?

What was the typical age at which girls got married back home in Syria?
Why do you think age at marriage was different in Syria?

- 1. **Suppose I am a young woman who got married at a young age. Are there any advantages to being married young?**

**Probe**

What are economic advantages of marrying early?

What are some social advantages (social status, education, social life/friends)?

What are health-related advantages?

Does marriage offer protection to girls? How so?

- 1. **What are disadvantages that arise from getting married at an early age?**

**Probe**

What are economic disadvantages of marrying early?

What are examples of social disadvantage (how does CM impact a girls education, her ability to work)?

Health-related disadvantages?

What challenges do girls who marry at a young age face?

- 1. **Some parents may prefer to marry their girls off at an early age. Why do you think parents may want their girls to marry young?**

**Probe**

What might be economic reasons?

Are there any protection concerns that might be driving their decision? What kinds of concerns?

What role do religious beliefs play in the decision-making process?

Do parents acquire a different social status if their daughter is married? Please explain

Section 3: Marriage arrangements and decision-making

- 1. **In your community, who makes the decision whether a girl gets married or not? What role do girls play in the decision-making process?**

**Probe**

What role do parents play in this decision?

What role do grandparents play in this decision?

What role do other members of the community play in this decision?

How are these decisions made?

- 1. **How does the decision-making process change based on how old a girl is?**

**Probe**

Are they the same decision makers? Are they different?

Did these decision makers change after you came to Egypt?

- 1. **A lot of families require husbands to pay a bride price. Talk to us about bride price and how bride price is negotiated in your community**

**Probe**

Is bride price given at marriage in your community?

Was it given back when you were in Syria?

Is it the same as it used to be?

Who decides bride price?

How do you feel about bride price?

Section 3: Experiences of child marriage

- 1. **Tell us about a girl you know who got married before the age of 18. What were the circumstances that led to her marriage?**

**Probe**

How did she meet her husband?

What role did her parents and community play? Who were the decisionmakers involved?

Why do you think she got married at this age?

- 1. **In your opinion, what changes – positive and negative – occurred in her life as a result of her decision to get married young?**

**Probe**

Education

Work opportunities

Social consequences

Emotional consequences

Section 4: Local traditions and harmful practices

**4.1 What aspects of marriage do you think changed after you came to Egypt?**

**Probe**

Did the age at marriage change? How?

Did the profile of the husband change? How? (nationality / age / socioeconomic status/ cousin or not)

**4.2 How does the local context in Egypt affect marriage practices in your community?**

**4.3 Many Egyptians practice female genital cutting (FGC). Are you familiar with this phenomenon? Can you tell us what it is? What are your views about it?**

Closing

**5. When you think about your future, what’s the best future you imagine for yourself**
