# Supplementary material for: Drivers and consequences of child marriage in a context of protracted displacement: a qualitative study among Syrian refugees in Egypt
Source: BMC Public Health. 2021 Apr 7;21:674. doi: 10.1186/s12889-021-10718-8 (PMC8028254; doi:10.1186/s12889-021-10718-8)
Supplement: Supplementary file 2 — Additional file 2. In-depth interview guides. [file 12889_2021_10718_MOESM2_ESM.docx]

**Interview Guide for Unmarried Girls**

Topic 1: Introduction

1. I would like to start by hearing you describe what an average day is like for you as a girl living in this area?
   1. A lot of people in this region have had their daily lives affected by conflict. Could you tell me how experiencing conflict has affected any of these things you do every day?
2. When you think about your future, what is the best future you could imagine for yourself?
   1. How has experiencing conflict and coming to Egypt affected this future?

Topic 2: Perceptions around marriage and relationships

These next questions are about marriage. First, I’d like to ask you about marriage in general:

1. What is the perception in your community of when a girl is ready to get married?

Probe

- 1. Age
  2. Physical features
  3. Family
  4. Religious considerations
  5. Biological considerations
  6. Culture? Traditions? Origin?
  7. Household skills

1. Around what age do most girls in your community get married?
   1. How is this the same or different from before you came to Egypt?
2. In some Egyptians communities, it is expected that girls must undergo FGM before they get married. Is this true in your community?
   1. How would you describe the perception of this phenomenon in your community?
   2. Has this perception changed since coming to Egypt?

Now I’d like to ask you some questions about your plans for marriage.

1. When do you feel you will be ready to marry?
   1. What would you like to do in your life before you get married?
2. When you think about your future, how would marriage help you achieve these goals?
   1. How would it make achieving these goals more difficult?
3. If you were planning to get married, who do you think would be involved in making that decision?
   1. What considerations do you think these people will make in deciding on your marriage?

Probe:

Economic

Social

Religious

Who would facilitate the introduction between the bride and groom? Can you explain how this process takes place?

- 1. How are these considerations different from before you came to Egypt?

1. What characteristics are important for you to have in a potential husband?
2. What factors might make it difficult for a girl in your community to get married?

Topic 3: Experiences and decision-making

Now I’d like to ask you about the marriage experience.

1. How do you expect girls’ lives change after they get married?
   1. Probe: How do their responsibilities in the family or in the community change?
2. How do you expect marriage will affect achieving the goals for your life you mentioned earlier?
3. How does a girl’s life change after she gets married?
4. Probe: How do her responsibilities in the family or in the community change?
5. In some cases, married couples fight or show anger toward each other. Often times, this can mean physical harm. Do you see this practice happening among your peers who are married?
6. If so, what is the community perception around this practice?
7. Do you feel that the amount that married couples show violence toward each other has changed since you came to Egypt?

Topic 5: Childbearing

The next questions are about pregnancy and children

1. At what age do girls in your community generally start having children?
2. What do you think is necessary for a girl to do before she gets pregnant? (Probe: What would make a girl prepared to have children?)
3. Where do you go in your community to receive medical care?

Topic 6: Access to services

1. What services currently exist in this community for adolescent girls your age?
2. What services do you think would be important for adolescent girls your age?
3. Is there anything else you would like to share about your experiences as an adolescent girl living in this area?

**Interview Guide for Married Girls**

Topic 1: Introduction

1. I would like to start by hearing you describe what an average day is like for you as a married girl living in this area?
   1. A lot of people in this region have had their daily lives affected by displacement. Could you tell me how experiencing conflict has affected any of these things you do every day?
2. When you think about your future, what is the best future you could imagine for yourself?
   1. How has displacement affected this future?

Topic 2: Perceptions around marriage and relationships

These next questions are about marriage. First, I’d like to ask you about marriage in general:

1. What is the perception in your community in Egypt of when a girl is ready to get married?
   1. Age
   2. Physical features
   3. Family
   4. Religious considerations
   5. Biological considerations
   6. Culture? Traditions? Origin?
   7. Household skills
2. Around what age do most girls in your community get married?
   1. How is this the same or different from before you came to Egypt?

Topic 3: Experiences and decision-making

Now I’d like to ask you some questions about your marriage experience.

1. How old were you when you got married? Did you feel ready to get married at that age?
   1. Why did you feel (ready/not ready)
2. When you think about the time leading up to your marriage, who was involved in making that decision?
   1. What considerations did (insert people) make when deciding on your marriage?
   2. Probe:

Economic

Social

Religious

Who facilitated the introduction between the bride and groom? Can you explain how this process took place?

1. What characteristics are were considered important when choosing your husband?

Probe: Nationality, age, marriage status, SES

1. Now I’d like to hear about your marriage ceremony. Can you tell me about the event?

Probe: Who performed the ceremony? Where did it take place? How was the marriage registered?

Now I’d like to ask you a bit about married life.

1. Can you tell me about your husband?

Probe: How old is he? What does he do for work? Where is he from originally?

1. How did your life change after you got married?
   1. Probe: How did your responsibilities in the family or in the community change? How did your relationship with your family change?
2. What are the roles you feel you have to fulfill as a wife?
   1. How are these different from the activities of your peers who are not married? (Probe: Education, employment, social status, etc)
3. In some cases, married couples fight or show anger toward each other. Often times, this can mean physical harm. Do you see this practice happening among your peers who are married?
   1. If so, what is the community perception around this practice?
   2. Have you had any negative sexual experiences with your husband?
   3. Has the amount that married couples show violence toward each other has changed since you came to Egypt? If so, how?

Topic 4: Local context and harmful practices

1. Many Egyptians practice female genital mutilation (FGM). Are you familiar with this phenomenon? Can you tell us what it is? What are your views about it?
2. FGM can be viewed as a way to make girls more marriageable in Egypt. Have you heard of any Syrian girls who were asked to do this to get married? What do you think about this?

Probe

How have girls in your community responded to such requests?

How have parents responded to such requests?

Topic 5: Childbearing

These next questions are about pregnancy and children.

1. Do you have any children?
   1. How old were you when you had your first child?
2. Tell us about your experience with pregnancy?

Probe: What about it was easy? What was difficult?

1. Did you seek medical care for your pregnancy and delivery? If so, where did you seek this medical care?
   1. How did you feel about the care you received?

Topic 6: Access to services

1. What health services currently exist in this community for adolescent girls your age? (Probe: reproductive health services, GBV)
2. What other services do you think would be important for adolescent girls your age?
3. Is there anything else you would like to share about your experiences as a married adolescent girl living in this area?

**Interview Guide for Mothers**

Topic 1: Introduction

1. I would like to start by hearing you describe what an average day is like for you as a mother of a young girl in this area?
   1. A lot of people in this region have had their daily lives affected by displacement. Could you tell me how experiencing displacement has affected any of these things you do every day?
2. When you think about the future of your children, what would be the best future you could imagine for your daughter?
   1. How do you think experiencing displacement and moving to Egypt has affected this future?

Topic 2: Perceptions around marriage and relationships

These next questions are about marriage. First, I’d like to ask you about marriage in general:

1. What is the perception in your community of when a girl is ready to get married?

Probe

- 1. Age
  2. Physical features
  3. Family
  4. Religious considerations
  5. Biological considerations
  6. Culture? Traditions? Origin?
  7. Household skills

1. Around what age do most girls in your community get married?
   1. How is this the same or different from before you came to Egypt?
   2. What is the legal age at marriage in Egypt?
2. What are the necessary steps you would have to go through if you wanted your daughter to get married? (Probe: Who would have to be consulted? What exchanges (mahr) or ceremonies would have to take place)

Now I would like to ask you some questions about your beliefs regarding marriage.

1. When you think about your marriage experience, do you want your daughter to have a similar experience? Why? In what ways?

Probe:

- - - Age at marriage
    - No of children
    - Socioeconomic factors
    - Education
    - Preparedness for marriage
    - Relationship with husband

1. What characteristics are important for your daughter’s (future) husband to have?
   1. How have your considerations for these characteristics changed since coming to Egypt?
2. How would getting married help your daughter achieve the goals you discussed earlier? How would it make those goals more difficult?
3. How have your feelings regarding marriage, for yourself or your daughter, changed since experiencing displacement?

Topic 3: Experiences and decision-making

1. How old were you when you first married?
2. How did your life change after you got married?
   1. Probe: How did your responsibilities in the family or in the community change?
3. How did your daughter’s life change after she got married?/ How do the lives of young girls change after marriage?
   1. Probe: How did your responsibilities in the family or in the community change?
   2. How did her relationship with her family change?
   3. How will decision-making around her life change?
4. In some cases, married couples fight or show anger toward each other. Often times, this can mean physical harm. Do you see this practice happening among young married couples in this community?
   1. What is the community perception around this type of violence between couples? (Probe: Is it positive, negative, or not generally discussed)
   2. How are conflicts/disputes resolved?

Topic 4: Local context and harmful practices

1. In many communities, FGM is a prevalent phenomenon. Is this true in your community?
   1. How has the perception of female genital mutilation changed since you came to Egypt?
2. Do you know/ have you heard of any Syrians who have practiced FGM? If so, tell us about their experience.

Probe:

1. What influenced the decision?
2. Who where when?
3. At what age?
4. What are consequences (legal/ marriage dynamics/ health consequences + complications/
5. How does this change how she is perceived within her community and the Egyptian community.

Topic 5: Childbearing

Now I would like to ask you some questions about pregnancy and children.

1. At what age did you have your first child?
   1. Do you think this was an appropriate age to have your first child?
2. How many children do people in this community generally have?
   1. How has this changed since you came to Egypt?
   2. Why do you think this is the case?
3. What advice would you give your daughter if she wishes to delay or prevent early pregnancy?

Probe

Which method?

Where would she seek the service?

Topic 6: Access to services

1. Where would you advise your daughter to seek reproductive health services like antenatal care, family planning, delivery care?
2. Is there anything else you would like to share about your experiences in this community?
