# Supplementary material for: Drivers and consequences of child marriage in a context of protracted displacement: a qualitative study among Syrian refugees in Egypt
Source: BMC Public Health. 2021 Apr 7;21:674. doi: 10.1186/s12889-021-10718-8 (PMC8028254; doi:10.1186/s12889-021-10718-8)
Supplement: Supplementary file 3 — Additional file 3. Key-informant interview guides. [file 12889_2021_10718_MOESM3_ESM.docx]

**Interview Guide for Religious/Community Leaders**

Section 1: Introduction

**1.1 Please describe your involvement with the Syrian refugee community in Egypt**

- 1. **We are interested in learning about the practice of child marriage among Syrian refugee communities residing in Egypt. Can you explain norms and customs around marriage in Syria?**

Probe

- - 1. How do girls learn about relationships in Syria and how do they meet their prospective husbands?
    2. Who decides whether a girl is ready to get married?
    3. What factors are considered before a girl is deemed ready for marriage?
    4. Who facilitates the introduction between a girl and a potential husband?
    5. What are views around bride price in this community?
    6. How long did the marriage process typically take?
  1. **Can you comment on changes in marriage practices that have taken place due to the displacement of Syrian refugees to Egypt?**
     1. How has age at marriage been impacted?
     2. Are intermarriages common? Why/Why not?
     3. How well do girls get to know their husbands before marriage?
     4. Did the profile of husbands change? How? (nationality / age / socioeconomic status/ cousin?)
     5. How has facilitation of the introduction between a girl and a potential husband changed?

Section 2: Drivers and consequences of child marriage

- 1. **We are hoping to understand the current drivers of child marriage in Syrian communities. Can you tell us reasons why Syrian families may be inclined to marry off their daughters at an early age in Egypt?**

Probe

- - 1. What are economic advantages of marrying early?
    2. What are some social advantages (social status, education, social life/friends)?
    3. What are health-related advantages?
    4. Does marriage offer protection to girls? How so?
  1. **Are there any religious factors driving these decisions? If so, what are they?**
     1. If you were approached by someone who wants to marry their daughter at a young age, what advice would you give them and why?
  2. **In your experience, who would you consider are the primary decision-makers in this process?**

Probe

- - 1. What role do parents play in this decision?
    2. What role do grandparents play in this decision?
    3. What role do other members of the community play in this decision?
    4. How are these decisions made?
    5. How has decision-making been impacted by displacement?
  1. **In your experience, what are some of the challenges faced by girls who are married at a young age?**

Probe

- - 1. Is marriage registration a challenge to girls who marry early? How so?
    2. Do girls who marry at a young age face problems accessing health services?
    3. Are young girls more likely to experience IPV?
  1. **How have girls and families managed to cope with these challenges?**

Section 4: Local Context

- 1. **As you know, female genital mutilation is prevalent in Egypt. How have attitudes towards FGM changed among refugees since their arrival to Egypt?**
  2. **Tells us about a case of a Syrian refugee who was asked to undergo FGM or underwent FGM.**
     1. What were the reasons underlying this decision?

**Interview Guide for Humanitarians**

Section 1: Introduction

- 1. **Please describe your role at the organization in which you are currently working**
  2. **We are interested in learning about the practice of child marriage among Syrian refugee communities residing in Egypt. Can you describe the scale of this phenomenon?**

Probe

- - 1. How prevalent is this phenomenon?
  1. **Can you comment on changes in the marriage practices of Syrian refugees that may have taken place in recent years?**
     1. Has the phenomenon increased/decreased/been the same more or less?
     2. Are intermarriages common? Why/Why not?
     3. Has protracted displacement caused changes in marriage practices? How so?

Section 2: Drivers and consequences of child marriage

- 1. **We are hoping to understand the drivers of child marriage in Syrian communities. Can you tell us reasons why Syrian families may be inclined to marry off their daughters at an early age in Egypt?**

Probe

- - 1. What might be economic advantages to early marriage?
    2. How does child marriage impact social status of parents? Girls?
    3. How does disrupted education effect decisions around child marriage?
    4. In what ways do security concerns impact decision-making around child marriage?
    5. Are there religious reasons driving this decision?
    6. Are there protection concerns driving this decision? – talk abt marriage registration etc
  1. **In your experience, who would you consider are the primary decision-makers in this process?**

Probe

- - 1. What role do parents play in this decision?
    2. What role do grandparents play in this decision?
    3. What role do other members of the community play in this decision?
    4. How is the introduction between a girl and a potential husband facilitated?
    5. How are these decisions made?
    6. How has decision-making been impacted by displacement?
  1. **In your experience implementing programs targeting Syrian refugees, what are some of the challenges faced by girls who are married at a young age?**

Probe

- - 1. Is marriage registration a challenge to girls who marry early? How so?
    2. Do girls who marry at a young age face problems accessing health services?
    3. Are young girls more likely to experience IPV?

Section 3: Coping strategies and interventions/programs

- 1. **How have girls and families managed to cope with these challenges?**
  2. **Can you tell us about programs/interventions undertaken by your organization to prevent or mitigate the impact of child marriage?**

Probe

- - 1. How long have they been implemented?
    2. Who do these interventions or programs target?
    3. How do you measure the degree to which they have been successful?
    4. In your opinion, how well have they been implemented?

Section 4: Local Context

- 1. **What impact, if any, has the local context had on the marriage practices of Syrian refugees?**

Probe

- - 1. How have local marriage practices impacted age at marriage?
    2. How have local marriage practices impacted decision-making around marriage?
    3. How have local marriage practices impacted bride price?
    4. How have local marriage practices impacted gender roles?
  1. **As you know, female genital cutting is prevalent in Egypt. How have attitudes towards FGM changed among refugees since their arrival to Egypt?**
  2. **Tells us about a case of a Syrian refugee who was asked to undergo FGM or underwent FGM.**
     1. What were the reasons underlying this decision?

Section 1: Introduction

- 1. Please describe your role at this health facility

Probe

- - 1. How long have you been working at this facility? In this position?
    2. What are your primary responsibilities?
  1. We are interested in learning about the practice of child marriage among Syrian refugees and Egyptians. From your experience providing services in this area, can you tell us if child marriage is practiced by Syrian communities? Can you tell us if child marriage is prevalent among Egyptian communities in this area?

Probe

- - 1. Can you describe the scale of this phenomenon in your experience delivering services here?
    2. Can you talk about the drives of this phenomenon in this area

Section 2: Service Provision

- 1. Can you describe to us your experience providing services to married girls under the age of 18?
  2. Suppose a fifteen-year old girl walks into this health facility wishing to insert an implant or IUD. Can you describe the process that takes place before she is able to receive the service?
     1. Does she have to register and show her national ID/ passport?
     2. Does her age pose a problem?
     3. Does she need to be accompanied by her husband?
     4. Which physician would she be able to see and why?
  3. Suppose the same girl walks into this health facility seeking a delivery service. Can you describe the process that takes place before she is able to deliver in the facility?
     1. Does she have to register and show her national ID/ passport?
     2. Does her age pose a problem?
     3. Which physician would she be able to see and why?
     4. Would she need to be accompanied by her husband?
  4. After delivery, describe to us the process leading to birth registration and how this process looks like for someone under the age of 18.
  5. Are there any legal issues that you must consider before providing services to girls under the age of 18?

Section 3: Access

- 1. **What are the biggest health challenges faced by girls who marry under the age of 18?**
  2. **What services would you say are most needed by this group?**
  3. **What challenges do girls face as they try to access these services?**
  4. **How have girls and their families managed to cope with these challenges?**
  5. **Can you tell us about programs/interventions that you are familiar with that target young girls?**

Probe

- - 1. How long have they been implemented?
    2. Who do these interventions or programs target?
    3. In your opinion, how well have they been implemented?

Section 4: Local Context

- 1. **As you know, female genital cutting is prevalent in Egypt. In your experience providing services to Syrian refugees and Egyptians, can you describe the attitudes these communities have towards this phenomenon?**
     1. Have attitudes towards FGM become more favorable among Syrian refugees?
  2. **If you are familiar with any cases of refugees who have undergone FGM, tell us about the case**
     1. What were the reasons underlying this decision?
     2. Where can refugees go to undergo the procedure? Do they go to different places?
     3. What were the consequences of her decision to undergo FGM?
